# Comment: Acute Myocardial Infarction and Stage E Shock: Insights From the RECOVER III Study

**DOI:** 10.1016/j.jscai.2025.103598

**Published:** 2025-04-08

**Authors:** Harry B. Peled, Quyen Dau, Jacob C. Jentzer

**Affiliations:** a Providence St. Jude Medical Center, Fullerton, California; b Department of Cardiovascular Medicine, Mayo Clinic, Rochester, Minnesota

We read with interest the recent paper published in *JSCAI* “Acute Myocardial Infarction and Stage E Shock: Insights From the RECOVER III Study,”^1^ which describes the clinical trajectory and survival for patients with acute myocardial infarction with cardiogenic shock in Society of Cardiovascular Angiography & Interventions (SCAI) Shock Stage E who received Impella support. We applaud the authors for examining a high-risk cohort to identify potential predictors of improvement in shock over 24 hours. Although the baseline characteristics of this cohort reflect a high severity of illness that in many cases likely represents refractory cardiogenic shock (CS), we do note that the authors appear to classify all post cardiac arrest patients as SCAI Stage E, and these patients represent >80% of patients. We respectfully disagree with the contention that preceding cardiac arrest requiring cardiopulmonary resuscitation automatically classifies a patient as SCAI Shock stage E, as this is not consistent with the rationale that SCAI Shock statement provided for adding the A modifier for (comatose) postarrest patients.^2^

Although some prior studies have used this same strategy of assigning postarrest patients into SCAI stage E,^3^ we feel that conflation of prior cardiac arrest and refractory shock fails to separate the adverse prognosis related to potentially reversible refractory CS from that due to irreversible anoxic brain injury. Rapid reversal of the shock state and metabolic perturbations (eg, severe lactic acidosis accumulated during cardiopulmonary resuscitation) can occur after return of spontaneous circulation following cardiac arrest, distinguishing this condition from true refractory CS. For this reason, although the authors did require an additional factor to classify in-hospital cardiac arrest as stage E, an elevated lactate level may not accurately reflect the CS phenotype.

The reported hemodynamic data suggest possible overstaging. Prior to Impella, less than two-thirds of patients were on >1 vasoactive drug (doses not available), with an average mean arterial pressure >80 mm Hg; this may be more consistent with stage C or D.^4^ We are uncertain if these findings are applicable to a refractory CS cohort without preceding arrest, recognizing that most SCAI Shock stage E patients have prior arrest. What is clear is that failure to improve from stage E despite Impella and vasoactive drug support is a major adverse prognostic marker, potentially justifying escalation of hemodynamic support in patients without contraindications (as was required in one-quarter of “nonresponders”). We would therefore be hesitant to draw any cause-and-effect conclusions based on this well-conducted single-arm observational study in which baseline conditions could have affected both treatment strategies and clinical outcomes.
